# Genome-based model for differentiating between infection and carriage *Staphylococcus aureus*

**DOI:** 10.1128/spectrum.00493-24

**Published:** 2024-09-09

**Authors:** Jianyu Chen, Wenyin Du, Yuehe Li, Huiliu Zhou, Dejia Ouyang, Zhenjiang Yao, Jinjian Fu, Xiaohua Ye

**Affiliations:** 1 Laboratory of Molecular Epidemiology, School of Public Health, Guangdong Pharmaceutical University, Guangzhou, China; 2 Department of Laboratory Science, Maoming Hospital of Guangzhou University of Chinese Medicine, Guangzhou, China; 3 Guangzhou Women and Children’s Medical Center Liuzhou Hospital, Liuzhou, China; NHLS Tygerberg/Stellenbosch University, Cape Town, Western Cape, South Africa

**Keywords:** *Staphylococcus aureus*, genome-wide association study, genomic epidemiology, bacterial genomics, pathogenicity

## Abstract

*Staphylococcus aureus* (*S. aureus*) is a clinically significant opportunistic pathogen, which can colonize multiple body sites in healthy individuals and cause various life-threatening diseases in both children and adults worldwide. The genetic backgrounds of *S. aureus* that cause infection versus asymptomatic carriage vary widely, but the potential genetic elements (k-mers) associated with *S. aureus* infection remain unknown, which leads to difficulties in differentiating infection isolates from harmless colonizers. Here, we address the disease-associated k-mers by using a comprehensive genome-wide association study (GWAS) to compare the genetic variation of *S. aureus* isolates from clinical infection sites (272 isolates) with nasal carriage (240 isolates). This study uncovers consensus evidence that certain k-mers are overrepresented in infection isolates compared with carriage isolates, indicating the presence of specific genetic elements associated with *S. aureus* infection. Moreover, the random forest (RF) model achieved a classification accuracy of 77% for predicting disease status (infection vs carriage), with 68% accuracy for a single highest-ranked k-mer, providing a simple target for identifying high-risk genotypes. Our findings suggest that the disease-causing *S. aureus* is a pathogenic subpopulation harboring unique genomic variation that promotes invasion and infection, providing novel targets for clinical interventions.

**IMPORTANCE:**

Defining the disease-causing isolates is the first step toward disease control. However, the disease-associated genetic elements of *Staphylococcus aureus* remain unknown, which leads to difficulties in differentiating infection isolates from harmless carriage isolates. Our comprehensive genome-wide association study (GWAS) found consensus evidence that certain genetic elements are overrepresented among infection isolates than carriage isolates, suggesting that the enrichment of disease-associated elements may promote infection. Notably, a single k-mer predictor achieved a high classification accuracy, which forms the basis for early diagnostics and interventions.

## INTRODUCTION

*Staphylococcus aureus* (*S. aureus*) is both a common commensal becterium and a clinically significant opportunistic pathogen, causing various diseases ranging from minor skin infections to severe invasive diseases such as bacteremia and septicemia (1, 2). According to the systematic analysis for global mortality associated with 33 bacterial pathogens (3), *S. aureus* was the leading bacterial cause of death and was associated with the most deaths in adults. More importantly, *S. aureus* has been the leading cause of hospital-associated infection and one of the most common causes of bloodstream infection in children worldwide (4), which is associated with significant morbidity, mortality, and hospital costs. Thus, *S. aureus* remains a challenging global health threat. Although *S. aureus* can colonize multiple body sites of healthy humans, the anterior nares are considered to be the most important colonization site (5, 6). Notably, the prevalence of *S. aureus* nasal carriage has been reported as high as 20%–30% (2, 7), with significantly higher rates in children ranging from 45% to 70% (5). *S. aureus* has become an urgent global public health priority because of its high carriage and pathogenicity in children. To improve the treatment and prevention of *S. aureus* disease, it is important to understand its pathogenesis.

According to clinical symptoms and pathogenicity, human-associated *S. aureus* is usually classified as disease-causing infection and asymptomatic carriage isolates. Latest evidence has revealed that bacterial pathogens (such as *Staphylococcus epidermidis*, *Streptococcus pneumonia*, and avian *Escherichia coli*) isolated from infections are a subset of the asymptomatic commensal population (8–10), indicating that there are specific clones or pathogenicity elements associated with pathogen evolution from harmless ancestors as well as promoting adhesion, migration, and invasion. To date, in China, because of the rarity of *S. aureus* carriage isolates from asymptomatic individuals, many previous studies have focused on clinical isolates of *S. aureus* infections, aiming to reveal and compare molecular genetic characteristics between different clinical clones (11). It remains unknown whether *S. aureus* infection isolates represent a unique pathogenic subgroup genetically different from asymptomatic carriage isolates. Moreover, the potential genetic elements associated with *S. aureus* infection remain poorly understood, which leads to difficulties in differentiating infection isolates from harmless colonizers.

**Fig 1:**
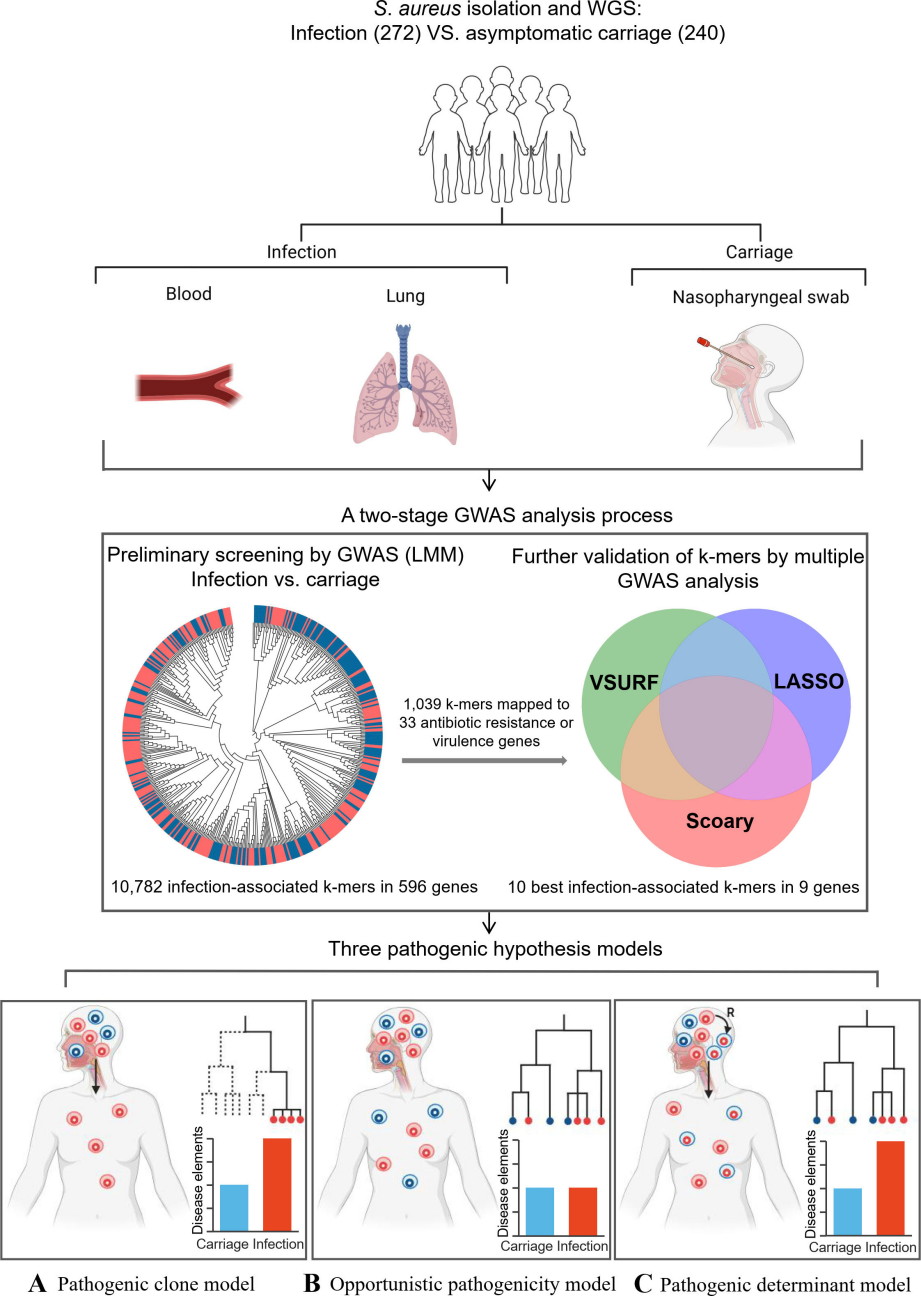
Characteristics and genetic relatedness of the *S. aureus* isolates used in the study. Potential pathogenicity models for *S. aureus* infections: (A) pathogenic clone model; (B) opportunistic pathogenicity model; (C) pathogenic determinant model.

In order to address the complex pathogenicity nature, there is a pressing need to consider different potential models for *S. aureus* infection based on the pathogenic clones and genome variation. First, a pathogenic clone model, in which only specific pathogenic clones with genomes enriched for disease-associated elements can cause infection (Fig. 1A). Second, a true opportunistic pathogenicity model, in which all clones are equally capable of causing infection (Fig. 1B). Third, a pathogenic determinant model (8, 10), in which enrichment of disease-associated elements may increase the risk of *S. aureus* pathogenicity, allowing divergent clones to successfully cause infection (Fig. 1C). Therefore, a genome-level comparison of the genetic differences between *S. aureus* infection and carriage isolates is urgently needed to reveal disease-associated genetic elements of *S. aureus*.

Traditional genotyping techniques (e.g., multi-locus sequence typing, pulsed-field gel electrophoresis, and *spa* typing) have been widely used to describe bacterial relatedness (12, 13), but they lack the discriminatory power for distinguishing small genetic differences between carriage and infection isolates. With the growing accessibility as well as falling costs, whole-genome sequencing (WGS) with its markedly high discriminatory power has become an important tool for the rapid generation of high-dimensional genomic data. Genome-wide association studies (GWAS) have played an increasingly important role in revealing the statistical link between genomic variation and bacterial phenotypes such as disease status, antimicrobial resistance, host adaptation, and virulence (8–10, 14, 15). To avoid the limitations of traditional GWAS based on single nucleotide polymorphisms (SNPs), we used k-mers (DNA words of length k) as an alternative method, which can capture different types of variants such as SNPs, genes, and insertions/deletions (16). Therefore, the application of k-mer-based GWAS to analyze bacterial genome variation has the potential to identify novel disease-associated genetic elements without annotation in the reference genomes, which may provide new clues for infection prevention and control strategies.

In order to combat this opportunistic pathogen, there is a pressing need to monitor genetically diverse commensal *S. aureus* clones and identify risk isolates that are prone to pathogenicity because of carrying disease-associated genetic elements. Therefore, we performed two-stage multiple GWAS analyses to compare the genomic differences between infection and asymptomatic carriage *S. aureus* isolates, so as to determine whether the presence of specific genetic determinants (k-mers) is disproportionately enriched in the infection isolates, which promotes its ability to cause disease. This study gains novel genome-wide insights into bacterial phenotypes, which may provide genetic evidence of tracing the highly pathogenic clones and designing precise clinical interventions.

## MATERIALS AND METHODS

### Bacterial sampling and identification

Liuzhou is a medium-sized economic city with a focus on industry development. This study was conducted in Liuzhou City, Guangxi Province, China, from 2014 to 2018 to collect 512 non-repetitive *S. aureus* isolates, including 272 infection and 240 asymptomatic carriage isolates from urban and suburban areas without proximity to livestock farms. Infection isolates were retrieved from the clinical specimens (e.g., sputum, abscess, blood, bronchoalveolar lavage fluid, and so on) of infected children in hospitals. Eligibility criteria for *S. aureus*-infected children were follows: (i) aged less than 7 years; (ii) having at least one infection symptom or sign (such as respiratory secretions, cough, dyspnea, sweating, temperature >38℃, and/or chest X-ray infiltrates); and (iii) providing clinical specimens from which *S. aureus* was isolated and positively cultured. Asymptomatic carriage isolates were retrieved from nasal swab specimens of healthy children aged less than 7 years from six kindergartens, including three kindergartens from urban areas and three kindergartens from suburban areas. All *S. aureus* isolates included in this study were not associated with known *S. aureus* outbreaks.

Methods of *S. aureus* identification were described in detail elsewhere (17). Briefly, *S. aureus* identification was performed based on a combination of colony morphology, gram staining reaction, *β*-hemolysis, catalase test, and tube coagulase test. After completion of diagnostics, *S. aureus* isolates were collected by the researchers and were stored at –80°C for further analysis.

### Genomic DNA extraction and sequencing analysis

The genomic DNA of all *S. aureus* isolates was extracted using the Magen Hipure Bacterial DNA Kit after incubation on broth medium for 24 hours at 37℃. The quality of the DNA was assessed with a Qubit fluorometer 2.0. The DNA samples that met the quality criteria were used for library construction. Sequencing libraries were generated using a TruSeq DNA Sample Preparation Kit (Illumina, USA). Then, a high-throughput genome sequencing was performed on the HiSeq 2000 machine (Illumina, San Diego, CA, USA) to obtain paired-end 150-bp reads. The quality control of raw reads was assessed using FastQC v0.11.5, and low-quality reads (a quality score of <30 and a length of <50 bp) and adapter regions were trimmed using Trimmomatic v0.36. Trimmed reads of each genome were assembled by SPAdes v3.6.1. The low-level contamination and species identification were checked using Kraken v1.1.1 (http://ccb.jhu.edu/software/kraken/). Based on the allelic profile of seven housekeeping genes of *S. aureus*, sequence types (STs) were inferred by comparing with the multilocus sequence typing (MLST) database. Clonal complexes (CCs) were determined using the eBURST algorithm (18).

### Phylogenetic analyses

Variant sites with SNPs were generated by mapping reads to a complete reference genome MRSA252 (GenBank accession: NC_002952) using Snippy v4.4.5 (https://github.com/tseemann/snippy) with default options. After removing recombination regions using Gubbins v2.4.1 (19), the recombination-free maximum likelihood phylogenetic tree based on core SNPs was constructed by FastTree v2.1.10 (20), using a GTR+Gamma model and 100 bootstrap replicates. The phylogenetic tree was visualized and annotated using ChiPlot (https://www.chiplot.online/).

### Counting and annotating k-mers

The k-mer-based, alignment-free method was applied to determine the genomic variation associated with *S. aureus* infection. All k-mers with lengths of 9–100 bp were extracted using fsm-lite (https://github.com/nvalimak/fsm-lite), which excludes all non-informative k-mers and then filters low-frequency k-mers to obtain k-mers with allele frequencies of 1%–99% (21). To annotate the identified k-mers using BWA-MEM v0.7.17 (22), each sequence was mapped to hyper-virulent and widely used *S. aureus* reference genomes (RF122, MRSA252, M013, NCTC8325, MSSA476, Mu3, Newman, N315) obtained from the Virulence Factor Database (http://www.mgc.ac.cn/VFs) and previous studies. Gene ontology annotations (GOs) were determined using the UniProt (https://beta.uniprot.org/).

### Two-stage GWAS analysis of disease-associated k-mers

In GWAS analysis models, we used *S. aureus* disease status (infection or carriage) as outcome variable and the k-mers matrix (presence or absence) as independent variable so as to explore the complex nonlinear association between k-mers and disease phenotypes. Due to high-dimensional genomic data, a two-stage analysis process was performed to identify the disease-associated k-mers by multiple GWAS methods including the linear mixed model (LMM), the phylogeny-based method (Scoary), the regularized regression model (least absolute shrinkage and selection operator, LASSO), and the machine learning method (random forest, RF) (23–26). In the first stage, a k-mers-based GWAS analysis based on LMM was performed to initially screen significant k-mers using Pyseer v1.3.10 (24), and a similarity kinship matrix based on the core genome phylogeny was constructed to correct for population structure. In the second stage, three GWAS methods (Scoary, LASSO, RF) were used to identify consensus significant k-mers, which may minimize false-positive associations and redundancy of k-mer predictors. Bonferroni correction (*α*/*N*) was used to control for false-positive rates due to multiple comparisons of k-mers (27). Scoary v1.6.16 (https://github.com/AdmiralenOla/Scoary) is a software designed to efficiently analyze potential associations between the presence or absence of biomarkers and observed phenotypes. LASSO regression, using the glmnet package in R v.4.1.3, is suitable for high-dimensional and high-correlated genome data, which can reduce the model complexity by compressing the coefficients of variables to zero (25). In the RF model, the variable selection using random forests (VSURF) in R v.4.1.3 was used to perform a two-step variable selection procedure, including interpretation (this is to rank and find important variables according to variable importance) and prediction (this is to use a stepwise forward strategy for variable selecting based on the smallest out-of-bag error) (28). The importance of each k-mer predictor was sorted by the mean decrease in impurity (mean decrease Gini, MDG). We used accuracy, sensitivity, specificity, positive predictive value, negative predictive value, kappa value, and receiver operating characteristic (ROC) curve to evaluate the predictive effect of each model.

## RESULTS

### Characteristics of *S. aureus* isolates

In this study, we analyzed 512 *S*. *aureus* isolates, including 272 infection isolates from children with infection disease and 240 carriage isolates from healthy children (Fig. 1), which were collected from 2014 to 2017 in Guangxi Province. Of the 272 infection isolates, 10 invasive isolates were obtained from blood, bronchoalveolar lavage fluid, and ascites, and 262 noninvasive isolates were obtained from sputum, abscess, and other specimens. We identified 60 STs belonging to 20 CCs inferred from whole-genome sequences. The most common genotypes based on STs were ST188 (*n* = 49), ST45 (*n* = 49), ST59 (*n* = 47), ST6 (*n* = 41), ST338 (*n* = 39), ST7 (*n* = 37), and ST5 (*n* = 36). The most prevalent CCs were CC59 (*n* = 90), CC45 (*n* = 51), CC188 (*n* = 50), CC5 (*n* = 49), CC6 (*n* = 43), CC7 (*n* = 39), and CC121 (*n* = 27; Table 1).

**TABLE 1:** Association analysis between predominant genotypes and disease status^*a*^

Genotypes (*n*)	Infection isolates(*n* = 272）	Carriage isolates(*n* = 240）	*χ* ^2^	*P*	OR (95% CI)
CC59(90)	51(18.8)	39(16.3)	10.54	<0.001	2.17 (1.35–3.49)
ST59(47)	15(5.5)	32(13.3)	9.35	<0.001	0.38 (0.20–0.72)
ST338(39)	32(11.7)	7(2.9)	14.19	<0.001	4.44 (1.92–10.25)
CC45(51)	9(3.3)	42(17.5)	28.63	<0.001	0.16 (0.08–0.34)
ST45(49)	7(2.5)	42(17.5)	32.82	<0.001	0.13 (0.06–0.28)
CC188(50)	39(14.3)	11(4.6)	13.77	<0.001	3.49 (1.74–6.97)
ST188(49)	38(13.9)	11(4.6)	12.98	<0.001	3.38 (1.69–6.78)
CC5(49)	27(9.9)	22(9.2)	0.09	0.771	1.09 (0.60–1.97)
ST5(36)	22(8.0)	14(5.8)	0.99	0.319	1.42 (0.71–2.84)
ST950(7)	0(0.0)	7(2.9)	6.03	0.014	0.09 (0.00–0.60)
CC6(43)	27(9.9)	16(6.7)	1.76	0.184	1.54 (0.81–2.94)
ST6(41)	26(9.5)	15(6.3)	1.90	0.169	1.59 (0.82–3.07)
CC7(39)	27(9.9)	12(5.0)	4.40	0.036	2.09 (1.04–4.23)
ST7(37)	26(9.5)	11(4.6)	4.71	0.030	2.20 (1.06–4.56)
CC121(27)	16(5.9)	11(4.6)	0.43	0.512	1.30 (0.59–2.86)
ST121(11)	5(1.8)	6(2.5)	0.27	0.606	0.73 (0.22–2.42)
ST946(7)	2(0.7)	5(2.1)	0.86	0.353	0.35 (0.07–1.81)
CC1(22)	11(4.6)	11(4.6)	0.09	0.764	0.88 (0.37–2.06)
ST1(16)	6(2.2)	10(4.2)	1.62	0.203	0.52 (0.19–1.45)

^*a*^ Data are presented as no. (%) or as otherwise indicated. CI, confidence interval; OR, odds ratio.

### Association between genotypes and disease status

**Fig 2:**
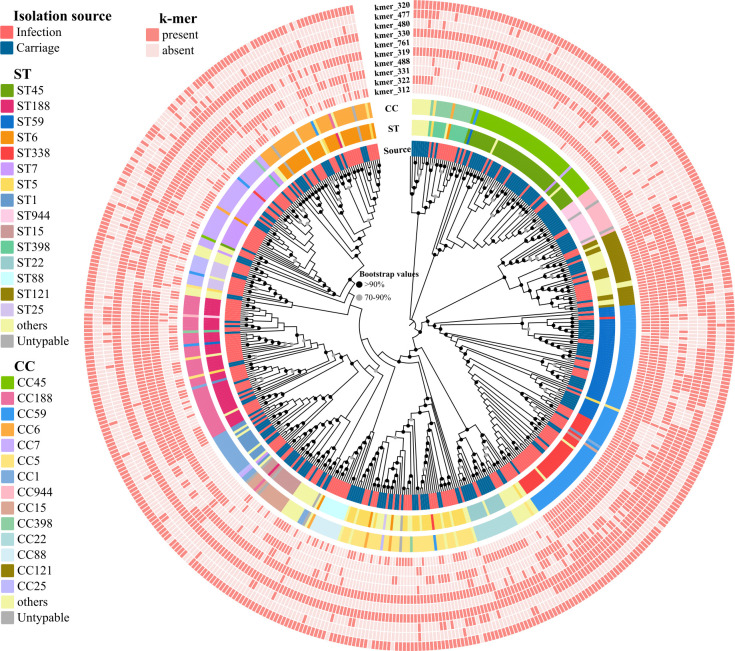
Whole-genome phylogenetic tree showing genetic similarity of the 512 *S*. *aureus* isolates. The color strips at the trip of the tree (from inner to outer) represent isolate metadata (isolation source, ST, and CC) and infection-associated k-mers found in the final model.

In terms of CC genotypes (Table 1), there were significant differences in the proportion of specific CCs (CC59, CC45, CC188, and CC7) between infection and carriage isolates (all *P* < 0.05), with significantly higher rates of CC59, CC188, and CC7 in infection isolates than in carriage isolates. In terms of ST genotypes, we also observed significant differences in the proportion of specific STs (ST59, ST338, ST45, ST188, ST950, and ST7) between infection and carriage isolates (all *P* < 0.05), with higher rates of ST338, ST188, and ST7 in infection isolates than in carriage isolates. This implied that specific genotypes may be associated with causing infection. Moreover, it was clear from the phylogenetic tree based on the core SNPs (Fig. 2) that the infection isolates did not represent a few pathogenic clones but clustered with carriage isolates in the same clades, indicating that there is a similar genetic background between two groups of isolates. The random forest models for predicting disease status (infection vs carriage) reached a classification accuracy of 68% based on 20 CC genotypes (63% for sensitivity and 77% for specificity) and 74% based on 60 ST genotypes (73% for sensitivity and 77% for specificity).

### Preliminary screening for infection-associated k-mers by LMM

**Fig 3:**
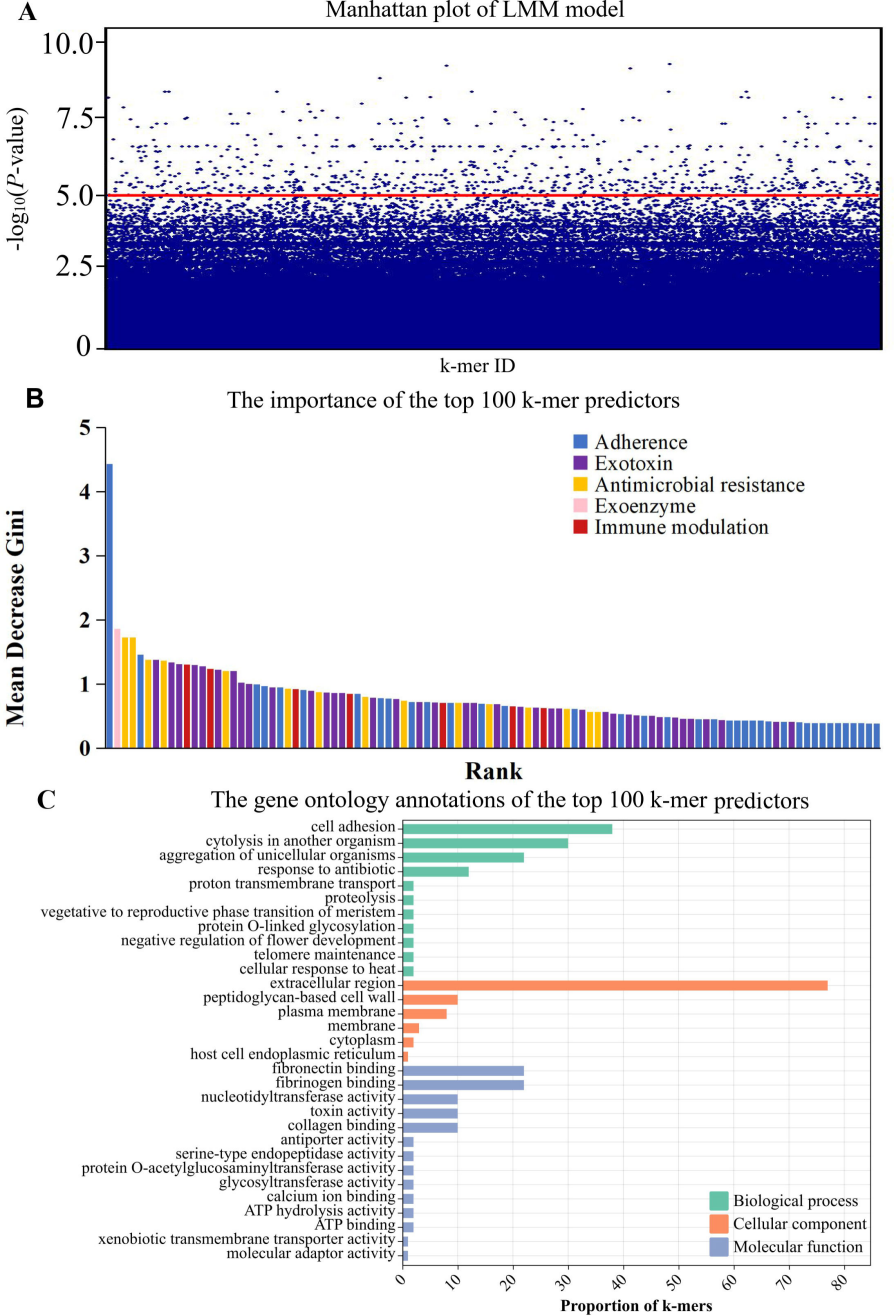
Preliminary screening for infection-associated k-mers by LMM. (**A**) Manhattan plot showing statistical significance in LMM-based GWAS. (**B**) Importance of the top 100 k-mer predictors in a simpler model with 1,039 k-mers. (**C**) The gene ontology annotations of the top 100 k-mer predictors.

We identified 19,828,368 k-mers from the genome sequences of 512 *S*. *aureus* isolates. After filtering out low-frequency k-mers (a minor allele frequency of <1%), we were left with 2,788,503 k-mers for the k-mers-based GWAS analysis (Fig. 3A). In order to reveal the genetic association between k-mers and disease phenotypes (infection or carriage), we used the LMM method (adjusted *P* value threshold of 1 × 10^−5^) to identify 12,178 significantly infection-associated k-mers after controlling for population structure, with 10,782 infection-associated k-mers successfully mapped to 596 genes with known functions. The resulting quantile–quantile (QQ) plot comparing the observed and expected *P* values confirmed adequate control of the population structure in the LMM-based GWAS. To account for considerable redundancy among the k-mer predictors, we used a simple model with only infection-associated 1,039 k-mers mapped to 33 antibiotic resistance or virulence genes to achieve a more accurate risk prediction (Table 2), with the classification accuracy being 80% (Table 3) and the area under the curve (AUC) value being 0.85, indicating that the predictive power of the simple model with only 1,039 k-mers is comparable with the complex model with 10,782 k-mers.

**TABLE 2:** Gene function annotation containing disease-related k-mers

Genes	k-mers hits	Max log (*P*)	Mean OR	Annotations
*cna*	78	6.24	1.67	Collagen adhesin precursor
*sei*	1	6.30	1.64	Staphylococcal enterotoxin type I
*clfB*	18	7.61	1.63	Clumping factor B, fibrinogen-binding protein
*tetM*	2	5.51	1.63	Affects the tetracycline resistance
*clpL*	2	5.17	1.62	Affects the disinfectant resistance
*sdrC*	165	7.61	1.57	Ser-Asp-rich fibrinogen-binding bone sialoprotein-binding protein
*ebh*	7	5.77	1.57	Hyperosmolarity resistance protein Ebh
*seu*	1	6.01	1.56	Staphylococcal enterotoxin type U
*sdrE*	71	10.71	1.55	Ser-Asp-rich fibrinogen-binding bone sialoprotein-binding protein
*adsA*	1	5.36	1.54	Adenosine synthase A
*fnbA*	126	7.14	1.52	Fibronectin-binding protein A
*norA*	1	6.63	1.52	Affects the fluoroquinolone resistance
*cap8K*	1	5.62	1.51	Type 8 capsular polysaccharide synthesis protein Cap8K
*seq*	103	5.74	1.50	Staphylococcal enterotoxin type Q
*hlgB*	5	5.67	1.46	Gamma-hemolysin component B
*splA*	4	5.33	1.43	Serine protease SplA
*lukM*	5	5.59	1.43	Bi-component leukocidin LukMF' subunit M
*mecA*	64	5.38	1.42	Affects the beta-lactamase resistance
*mecR*	2	4.83	1.42	Affects the methicillin resistance
*fnbB*	2	5.92	1.42	Fibronectin-binding protein B
*sek*	10	6.69	1.41	Staphylococcal enterotoxin type K
*sasA*	6	5.84	1.40	Serine-rich repeat glycoprotein adhesin SasA
*sep*	5	5.62	1.40	Staphylococcal enterotoxin type P
*lukS-PV*	233	10.21	1.39	Panton-Valentine leukocidin chain S precursor
*lukF’*	100	5.14	1.39	Bi-component leukocidin LukMF' subunit F'
*lukF-PV*	94	7.63	1.39	Panton-Valentine leukocidin chain F precursor
*lukE*	1	5.20	1.39	Bi-component leukocidin LukED subunit E
*sdrM*	1	5.77	1.38	Ser-Asp-rich fibrinogen-binding bone sialoprotein-binding protein
*seg*	1	5.69	1.36	Staphylococcal enterotoxin type G
*hlgA*	1	7.59	1.35	Gamma-hemolysin chain II precursor
*hlgC*	1	4.86	1.32	Gamma-hemolysin component C
*aadD*	8	6.15	1.31	Affects the aminoglycoside resistance
*sec*	1	4.91	1.31	Staphylococcal enterotoxin type C

**TABLE 3:** Resubstitution estimate and cross-validation results based on random forest models^*a*^

Evaluation indicators	1,039 k-mer predictors		10 k-mer predictors	
	Resubstitution estimate	Tenfold cross-validation	Resubstitution estimate	Tenfold cross-validation
Accuracy	0.92	0.80	0.81	0.77
Sensitivity	0.92	0.79	0.80	0.78
Specificity	0.92	0.82	0.83	0.78
PPV	0.93	0.85	0.86	0.82
NPV	0.90	0.74	0.76	0.74
Kappa	0.84	0.60	0.62	0.55

^*a*^ PPV, positive predictive value; NPV, negative predictive value.

Among the 33 functional genes associated with k-mer hits (Table 2), the largest average effect was observed in the *cna* gene (OR = 1.67), followed by *sei* (OR = 1.64), *clfB* (OR = 1.63), *tetM* (OR = 1.63), *clpL* (OR = 1.62), *sdrC* (OR = 1.57), *ebh* (OR = 1.57), *seu* (OR = 1.56), *sdrE* (OR = 1.55), *adsA* (OR = 1.54), *fnbA* (OR = 1.52), and *norA* (OR = 1.52), indicating the importance of relative abundance of specific k-mers in infection strains. The importance of the 100 highest-ranked k-mers is illustrated in Figure 3B, indicating that these k-mers were mainly associated with adherence, antimicrobial resistance, exotoxin, exoenzyme, and immune modulation. In addition, these top 100 k-mers annotated with GO terms were divided into three functional GO categories. The biological process mostly enriched in cell adhesion, the cellular component significantly enriched in extracellular region, and the molecular function mainly enriched in fibronectin and fibrinogen (Fig. 3C).

### Further validation of infection-associated k-mers by multiple GWAS analysis

**Fig 4:**
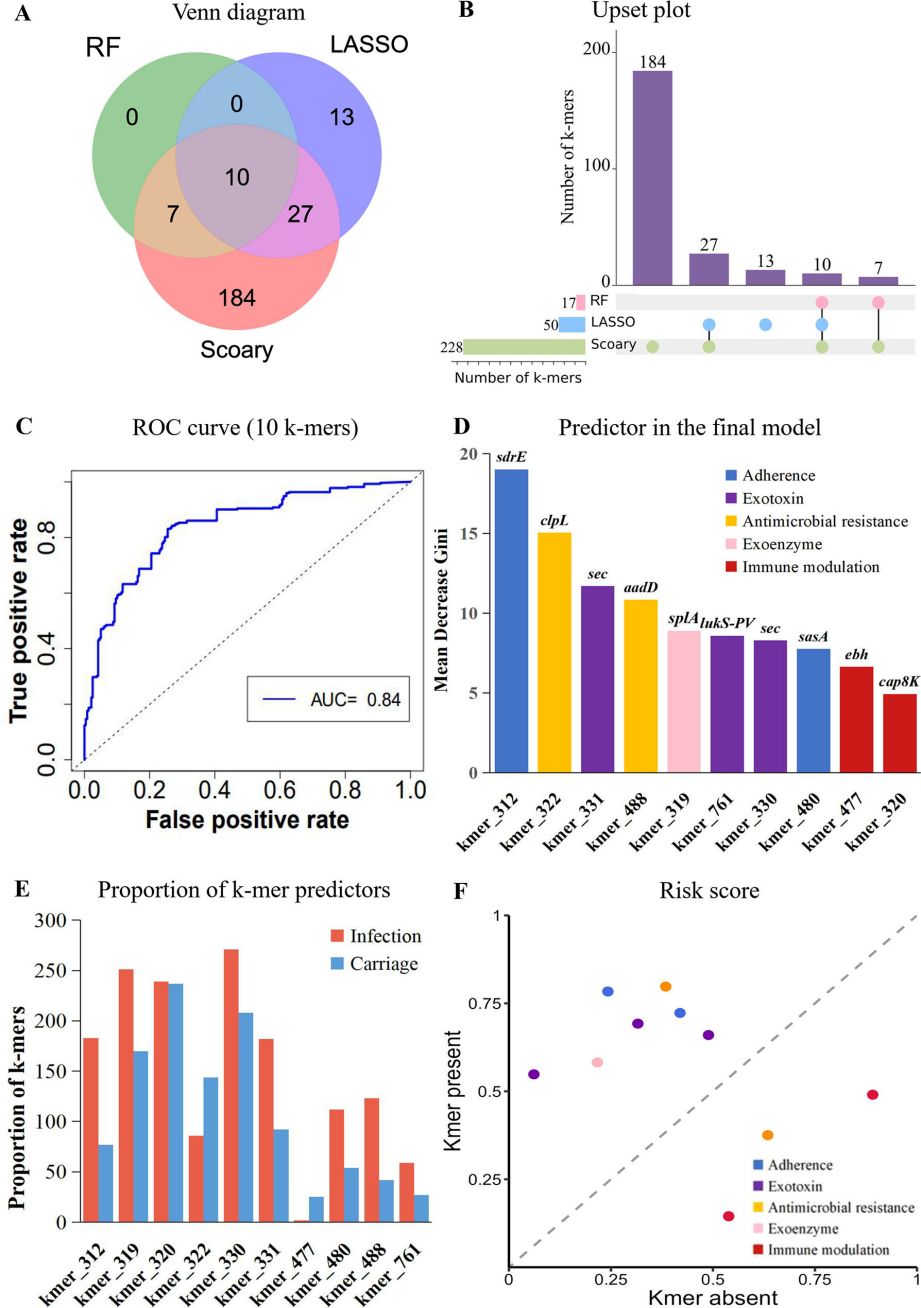
Further validation of infection-associated k-mers by multiple GWAS. (**A**) Venn diagram visualization of the k-mers identified by three methods. (**B**) UpSet plot visualization of the k-mers identified by three methods. (**C**) ROC curve showing the overall performance of the final model. (**D**) Predictor importance of the 10 k-mers included in the final model. (**E**) Proportion of the 10 k-mers between infection and carriage isolates. (**F**) Change in risk score for a specific k-mer profile when the color-indicated k-mer is present (*y*-axis) compared to absent (*x*-axis).

To minimize false-positive associations and reduce the complexity of the prediction model based on 1,039 pathogenicity-associated k-mers, three methods were performed to further screen the pathogenicity-associated k-mers. According to the Venn diagram and UpSet plot (Fig. 4A and B), multiple GWAS analyses based on 1,039 k-mer indicated consensus statistically significant associations with the disease status and identified 10 consensus k-mers associated with infection, suggesting that certain disease-associated elements are overrepresented in infection than in carriage isolates. For the final model with 10 consensus k-mers, the classification accuracy reached 77% (78% for sensitivity and 78% for specificity; Table 3) , with the AUC value being 0.84 (Fig. 4C), suggesting that the predictive power of the simple model with only 10 k-mers is close to the complex model with 1,039 k-mers.

The importance of disease-associated k-mers identified in the final model revealed that these k-mer predictors were associated with adherence (Kmer_312 in *sdrE*; Kmer_480 in *sasA*), antimicrobial resistance (Kmer_488 in *aadD*; Kmer_322 in *clpL*), exotoxin (Kmer_330 or Kmer_331 in *sec*; Kmer_761 in *lukS-PV*), exoenzyme (Kmer_319 in *splA*), and immune modulation (Kmer_320 in *cap8K*; Kmer_477 in *ebh*) (Fig. 4D). Surprisingly, the best k-mer predictor associated with adherence (Kmer_312 in *sdrE*) had a classification accuracy of 68%, a specificity of 65%, and a sensitivity of 71%, suggesting that the predictive classifier based on a single k-mer is powerful. Additionally, an independent data set available on the NCBI database (60 infection vs 60 carriage isolates) was used to perform an additional validation analysis based on the best RF classifier (Kmer_312 in *sdrE*), with the classification accuracy being 71%, a specificity of 73%, and a sensitivity of 69%, which may have potential for use as infection biomarkers in a clinical setting.

We observed statistically significant differences in the prevalence of all k-mers between infection and carriage isolates (*P* < 0.005, Fig. 4E; Table 4), suggesting that seven k-mers are overrepresented in the infection than in the carriage isolates. Figure 4F shows the overall effect of each k-mer on the estimated risk score of the prediction model. A point above the diagonal indicates that the presence of a specific k-mer increases the risk score. Notably, the presence of k-mers associated with adherence (OR = 1.45 for Kmer_312 in *sdrE*; OR = 1.32 for Kmer_480 in *sasA*), exoenzyme (OR = 1.32 for Kmer_319 in *splA*), exotoxin (OR = 1.80 for Kmer_330 in *sec*; OR = 1.31 for Kmer_331 in *sec*; OR = 1.49 for Kmer_761 in *lukF-PV*), and antimicrobial resistance (OR = 1.39 for Kmer_488 in *aadD*) genes increased the risk of *S. aureus* infections (Table 4).

**TABLE 4:** Association analysis between k-mers and disease status^*a*^

K-mers	Genes	Infection isolates (*n* = 272)	Carriage isolates (*n* = 240)	*P* value	OR (95% CI)
Kmer_312	*sdrE*	183(67.2)	77(32.2)	1.94E-11	1.45 (1.39–1.50)
kmer_480	*sasA*	112(41.2)	54(22.5)	9.73E-06	1.32 (1.25–1.38)
Kmer_330	*sec*	271(99.6)	208(86.7)	3.42E-08	1.80 (1.70–1.91)
Kmer_331	*sec*	182(66.9)	92(38.3)	1.22E-05	1.31 (1.25–1.37)
Kmer_761	*lukS-PV*	59(21.7)	27(11.3)	6.80E-08	1.49 (1.42–1.56)
Kmer_488	*aadD*	123(45.2)	42(17.5)	2.80E-06	1.39 (1.32–1.46)
Kmer_322	*clpL*	86(31.6)	144(60.0)	6.70E-06	0.64 (0.54–0.74)
Kmer_319	*splA*	251(92.3)	170(70.8)	4.70E-06	1.32 (1.26–1.38)
Kmer_320	*cap8K*	239(87.9)	237(98.8)	2.42E-06	0.66 (0.58–0.75)
Kmer_477	*ebh*	2(0.7)	25(10.4)	2.50E-06	0.63 (0.53–0.73)

^*a*^ Data are presented as no. (%) or as otherwise indicated. CI, confidence interval; OR, odds ratio.

## DISCUSSION

Molecular typing has become the most common tool for describing bacterial relatedness and distinguishing pathogenic clones, thus providing evidence for revealing potential pathogenic mechanisms. In this study, the predominant lineages of all *S. aureus* isolates were CC59 (ST59 and ST338), CC45 (ST45), CC188 (ST188), and CC5 (ST5), with CC59 (ST338) and CC188 (ST188) as the most prevalent clones among the infection isolates. These results were consistent with the latest reports from China (11, 29, 30), but were different from findings in South America, Spain, and Egypt (31–33), suggesting considerable regional differences and genetic diversity. In this study, we observed significant associations between specific genotypes (such as CC59, CC45, CC188, and CC7) and disease status, suggesting that the presence of specific pathogenic clones may promote its ability to cause disease. However, traditional genotyping techniques with low discriminatory power cannot distinguish between closely related isolates from the same clone and is difficult to reveal small genetic differences between carriage and infection isolates, indicating that these genotypes only partially explain the pathogenicity of *S. aureus*. Strikingly, several clone lineages (e.g., CC45/USA600, CC5/USA100, CC1/USA400, and CC188), known as hyper-virulent clones, have circulated worldwide and are frequently associated with life-threatening invasive diseases or bloodstream infections (34–36). Surprisingly, these hyper-virulent clones were observed in both the infection and carriage isolates in our study, providing more evidence that traditional genotypes only partially explain the pathogenicity of *S. aureus* and specific genetic elements may also promote infection and invasion.

Considering the complex multifactorial nature of pathogenicity, high-throughput genome sequencing with high resolution is clearly an important tool for identifying novel genetic variants associated with bacterial phenotypes. Notably, the simple pathogenic clone model was not observed for *S. aureus* in this study as well as for *S. epidermidis* and *avian E. coli* in previous studies (8, 10). In this study, the phylogenetic tree based on core genome SNPs revealed that the infection isolates were not from specific pathogenic clones but clustered tightly in a highly uniform clade with carriage isolates of multiple genetic backgrounds, suggesting that most clones are equally capable of causing disease (that is, opportunistic pathogenicity model). If the opportunistic pathogenicity is suitable for *S. aureus* infections, disease-associated elements are equally enriched in infection and carriage isolates (no significant difference). However, in this study, the k-mer-based GWAS analysis using LMM identified numerous significant k-mers mapped to genes associated with pathogenicity. These findings suggest that pathogenic isolates are a subset of the asymptomatic commensal population and that the enrichment of disease-associated elements may promote invasion (that is, the pathogenic-determinant model). This is consistent with findings from *S. epidermidis* and *avian E. coli* (8, 10), suggesting that horizontal gene transfer spreads disease-associated genetic elements into multiple genomic backgrounds leading to the emergence of various pathogenic clones.

Previous bacterial GWAS studies have focused on a single method to reveal disease-associated variation of *S. aureus*, *S. epidermidis*, *avian E. coli*, and other bacterial pathogens (8, 10, 37). In order to reduce redundancy of genetic predictors and false-positive associations, we build on previous literature to use a two-stage comprehensive GWAS analysis to confirm the potential pathogenic-determinant model as well as obtain a simpler prediction model. Interestingly, we found consensus evidence that certain genetic elements (10 k-mers) are overrepresented in infection than in carriage isolates. The final prediction model based on a specific k-mer profile (10 k-mers) achieved a classification accuracy of 77%, offering a very simple target for identifying high-risk genotypes. These 10 consensus k-mers successfully mapped to genes associated with adherence (*sdrE*, *sasA*), antibiotic resistance (*aadD*, *clpL*), exotoxin (*sec*, *lukS-PV*), exoenzyme (*splA*), and immune modulation (*ebh*, *cap8K*), indicating the multifactorial nature of the pathogenicity. The pathogenic mechanism of *S. aureus* infection is a complex process that involves several key steps including adhesion to the surface, invasion of host cells, biofilm formation, and evasion of immune responses, and many of these steps are facilitated by important virulence factors such as cell surface components, enzymes, and exotoxins (38, 39). In the process of *S. aureus* pathogenicity, adhesion in host cells is the first and most important step, which is a prerequisite for biofilm formation as well as host cell invasion. *S. aureus* surface protein SdrE (encoded by *sdrE*), known as one of the most important cell wall-anchored proteins, can carry out multiple functions. For example, SdrE contributes to immune evasion by binding complement regulator factor H, thereby inhibiting complement activation and amplification to reduce bacterial killing by granulocytes; SdrE also takes part in promoting the destruction of C3b to decrease complement-mediated phagocytosis (40–42). Notably, in the present study, a single k-mer classifier based on the best k-mer predictor associated with adherence (Kmer_312 in *sdrE*) achieved a high classification accuracy of 68%, having the potential for the development of pathogenicity biomarkers in a clinical setting. *S. aureus* surface proteins A (*sasA*), known as an important group of cell surface adhesins, plays a crucial role in *S. aureus* virulence by mediating attachment to a variety of host cells and biofilm formation (43, 44). Of the exotoxin, the staphylococcal enterotoxin (SEC) is involved in at least three biological activities including superantigenicity, pyrogenicity, and lethality (45); Panton-Valentine leucocidin is the most important virulence factor of *S. aureus* encoded by *lukF-PV* and *lukS-PV*, which interferes with immune cells to cause cytokine release and cell death by apoptosis or necrosis (46, 47). The potential relationship between antibiotic resistance and pathogenicity in *S. aureus* remains unclear. However, we found two k-mer predictors associated with antimicrobial resistance (Kmer_488 in *aadD*; Kmer_322 in *clpL*), and a previous study revealed that the expression of high-level antibiotic resistance in *S. aureus* resulted in the repression of biofilm production (48), suggesting that the presence of specific antibiotic resistance genes and its tendency to form biofilms contribute to its pathogenicity. In summary, our findings suggest that the prediction model based on a specific k-mer profile forms the basis for clinical diagnostics and interventions.

To the best of our knowledge, this study is a new attempt to identify consistently significant genetic variation associated with *S. aureus* infections using a comprehensive GWAS analysis, thereby minimizing redundancy of k-mer predictors. There are some limitations to be taken into consideration. First, there is a transition from nasal carriage to infection states (49), thereby resulting in misclassification of isolate states. So, we collected carriage isolates from asymptomatic healthy children (not patients) and infection isolates from confirmed patients with infectious symptoms and signs (not healthy children), which can minimize the probability of misclassification and improve the statistical power. Second, strong population structure and genome-wide linkage disequilibrium, resulting from clonal transmission, are two main confounding factors in bacterial GWAS, leading to false-positive associations. So, we performed a robust LMM method to correct for population structure in terms of a similarity kinship matrix and collected isolates from a single location to minimize linkage disequilibrium resulting from geographical variation. In addition, we replicated the results using multiple GWAS methods to identify the consensus disease-associated k-mers, thereby minimizing false-positive associations. Third, although it is one of the large-sample studies on this topic, it only reflects an overall status of genomic variation of *S. aureus* isolates from one city in China. Future comparative genomics of disease and carriage *S. aureus* are required to validate our findings. Finally, although our GWAS analysis revealed specific pathogenicity-associated k-mers, further gene-based GWAS studies are required to confirm the effects of these significant k-mers.

In conclusion, our comprehensive GWAS analyses based on screening and validation found consensus evidence that certain genetic elements are overrepresented among infection isolates than asymptomatic carriage isolates, suggesting that pathogenic isolates are a subpopulation of the asymptomatic commensal isolates and that the enrichment of disease-associated elements may promote invasion and infection. Notably, both a certain k-mer profile and a single k-mer predictor achieved a high classification accuracy, which forms the basis for early diagnostics and interventions after further functional validation. Defining the disease-causing isolates is the first step toward disease control. Our study highlights the need to fully utilize GWAS findings to gain novel insights into the nature of bacterial pathogenicity and to inform medical interventions.

## Data Availability

The sequence data have been submitted to Sequence Read Archive database (https://www.ncbi.nlm.nih.gov/sra; BioProject nos. PRJNA1039716 and PRJNA1035931), and the clinical origins of strains and sequences of significant k-mers are publicly available (https://doi.org/10.6084/m9.figshare.26313022.v1).
